# Topography inversion in scanning tunneling microscopy of single-atom-thick materials from penetrating substrate states

**DOI:** 10.1038/s41598-022-10870-0

**Published:** 2022-05-05

**Authors:** Changwon Park, Mina Yoon

**Affiliations:** 1 School of Computational Sciences, Korea Institute for Advanced Study, Hoegiro 85, Seoul, 02455 Republic of Korea; 2 Materials Science and Technology Division, Oak Ridge National Laboratory, Oak Ridge, TN 37831 USA

**Keywords:** Condensed-matter physics, Surfaces, interfaces and thin films

## Abstract

Scanning tunneling microscopy (STM) is one of the indispensable tools to characterize surface structures, but the distinction between atomic geometry and electronic effects based on the measured tunneling current is not always straightforward. In particular, for single-atomic-thick materials (graphene or boron nitride) on metallic substrates, counterintuitive phenomena such as a larger tunneling current for insulators than for metal and a topography opposite to the atomic geometry are reported. Using first-principles density functional theory calculations combined with analytical modeling, we reveal the critical role of penetrating states of metallic substrates that surpass 2D material states, hindering the measurement of intrinsic 2D materials states and leading to topography inversion. Our finding should be instrumental in the interpretation of STM topographies of atomic-thick materials and in the development of 2D material for (opto)electronic and various quantum applications.

Scanning tunneling microscopy (STM) is a powerful tool that provides the topographic image and electronic structure of surface at atomic resolution. The topographic images obtained by mapping the tunneling current have provided numerous key data to characterize the details of the surface structures, including the surface reconstruction and the charge density wave^1–6^. Since the tunneling current is determined by both the electronic structure and the geometry of the surfaces, it is not always straightforward to determine true atomic structure of the surface from the apparent topographic image. One of the most commonly studied nontrivialities is the contrast inversion in the topography as a function of tip-to-sample distance, which is observed for various systems: Fe_2_N surface^7^, TiO_2_ surface^8^, and oxygen adatoms on metal surfaces^9–11^. These phenomena are on account of the multiorbital nature of the sample states, as the tunneling current is sensitive to the tails of the decaying wave functions of the sample in vacuum, whose decay rates are orbital dependent.

Complications also arise in the topography of geometrically corrugated atomic monolayer on metal surface, such as BN nanomesh on Ru^12,13^, Ir^14,15^, and Rh^16,17^ substrates and graphene nanomesh on Ir^18–21^ and Au^22–24^ substrates. STM topography of the periodic corrugations are inverted when the measuring condition changes as reported for STM^25–27^ and atomic force microscopy^28^ of BN on Rh(111) or graphene on Au(111). For BN monolayers, the inversions were explained by the bias-dependent electronic structure^26^ but in some case^29^, two inverted images occur simultaneously at the same bias. For graphene on Au(111), the simultaneous appearance of convex and concave topography is interpreted as true geometric corrugation^22^ based on the similar energy of both structures but it cannot explain the observed dominance of convex geometry.

In this paper, we reveal that the STM topography of an atomically thick material on a metal surface can be strongly affected by the substrate that the apparent relative height can be severely flattened or even opposite to the true atomic geometry, i.e., a higher location looks lower and vice versa. The phenomenon is quite general for insulators with one-atom thickness, where penetrating substrate states dominate the tunneling current. Unexpectedly, even in graphene, the penetrating substrate states readily supersede the intrinsic electronic structure of graphene, so cautions should be exercised in interpreting the topography data. Quantitative analysis of the penetration explains counterintuitive effects such as the larger tunneling current on the BN monolayer than on graphene and the general topography inversions for atomic monolayers. This suggests that a systematic study of tip-to-sample distance-dependent measurements is imperative for geometrically corrugated monolayers on metal surfaces or lateral heterostructures^30,31^.

For the STM simulation, we use Cu(111) surface as a noble metal substrate for atomic-thickness materials. The surface is modeled as a slab geometry consisting of 21 layers of Cu thick enough to avoid any artificial interaction between the top and bottom surface states. Our calculations show that at the $\Gamma$-point, the energy splittings (interaction strength) between top and bottom surface states decrease with the thickness of Cu layer: They are 69, 29 ,13 and 5 meV for 12, 15, 18 and 21 layers, respectively. We obtain optimized lattice constants of 2.467 (2.46), 2.512 (2.51), and 2.557 (2.56) Å for graphene, BN, and face-centered cubic Cu, respectively, which agree very well with the experimental values listed in the parentheses. We model two-dimensional (2D) materials as flat systems, i.e., any out-of-plane atomic corrugations that might be present under actual experimental conditions are neglected, with the lattice constant having an average value of 2.494 Å to keep the 2D systems on the substrate to be commensurate. Argon (benzene) monolayers are also constructed to be commensurate with 1 × 1 $\left( {\sqrt 7 \times \sqrt 7 } \right)$ of the Cu(111) surface. The local density of states (LDOS) are plane-averaged and we will use the terms integrated LDOS and tunneling current interchangeably.

Figure 1a illustrates the framework of our STM simulations. Here we consider the effect of two main parameters, the tip-to-2D material distances ($h$) and the 2D material-to-substrate distances ($d$). We ignore any tip effects by assuming that the tip states are spherically symmetric and energy-independent, so that the local density of states is proportional to the tunneling current according to Tersoff and Hamann^32^. Cu(111), graphene and BN are selected as prototypical models for metallic substrate, metallic and insulating 2D materials, respectively. Figure 1b and c shows the atomic structures of graphene and BN on top of the substrate, with a sublattice of graphene or nitrogen atoms of BN on Cu atoms. These 2D materials interact weakly with the substrate, i.e., in the sense that there are no chemical bonds between them (adsorption energy on the van der Waals scale) and their band structures remain almost intact upon the adsorption to the substrate. In Fig. 1d, the 2D band structure of graphene on Cu(111) (green) is compared with that of the Cu(111) surface (black dotted) and graphene (red) subsystems. The three band structures are calculated separately and plotted together with the corresponding energy shifts to match them. The only noticeable changes due to interaction are the electron doping on graphene (0.29 eV shift of Dirac point) and the energy shift of top surface states of Cu(111). Note that our main results do not rely on the specific lattice symmetry, that is, relative rotation, translation, or lattice mismatch between 2D materials and Cu(111) play a minor role.

**Figure 1:**
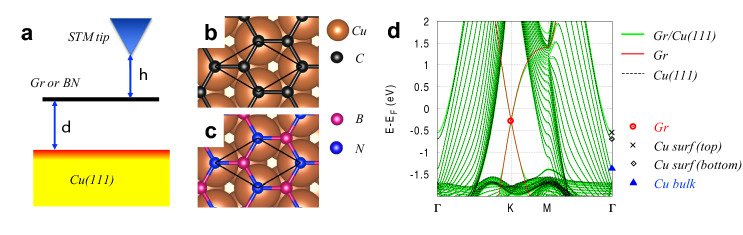
STM setup and weakly interacting graphene and boron nitride on Cu surface. (**a**) Schematic representation of the STM setup of a 2D material on Cu(111). Atomic structures of graphene (**b**) and boron nitride (**c**) on Cu(111) surface (surf). (**d**) Band structure of graphene on Cu(111) at ${\varvec{d}} = \user2{ }$ 3.3 Å (green), graphene (red), and bare Cu(111) (black dotted). The latter two band structures are shifted in energy to match the former. Four representative states at K and G points are indicated with symbols.

Next, we investigate the characteristics of the tunneling current as a function of $h$. Figure 2 shows the evolution of the plane-averaged LDOS and constant height current maps of Gr/BN on Cu(111) with $h =$ 2, 3 and 4 Å. Here, the LDOS are decomposed into Cu bulk (yellow), Cu surface (red) and graphene (black) based on their constituent wavefunction characters. Around 1 eV from the Fermi level, the Bloch wavefunctions of Cu and graphene are well distinguished (see Fig. 1d), while outside this range they start to mix and are presented as yellow-black graded colors. The criteria for the wavefunction characterization can be found in Sect. 1 of Supplementary Information. Corresponding constant-height current maps with a bias 0.5 V are shown in the right two columns of Fig. 2, with each current map is scaled by its average integrated LDOS ($\rho_{{{\text{av}}}}$) indicated on the image.

**Figure 2:**
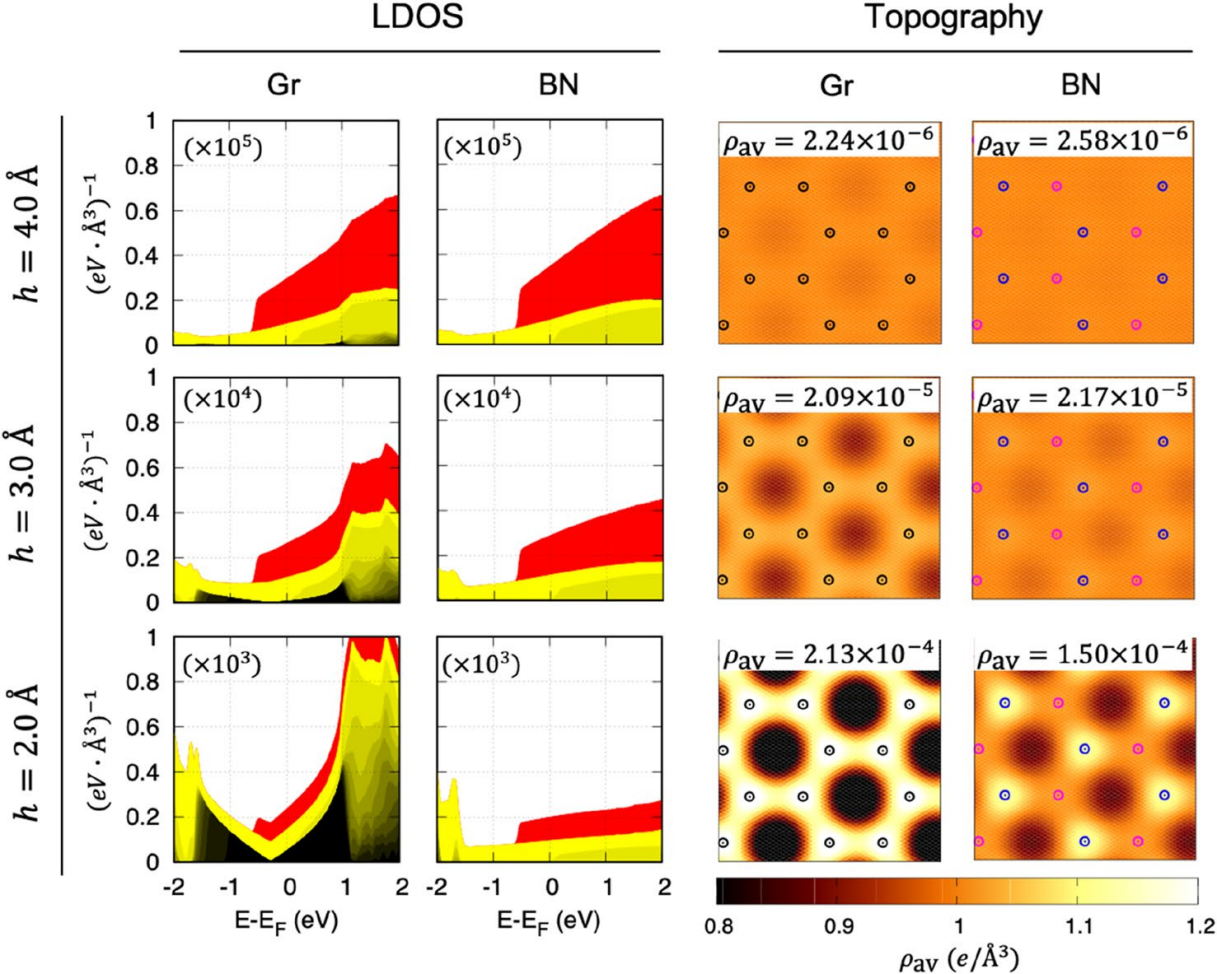
Tip height-dependent STM simulation of graphene and boron nitride on Cu surface. Local density of state (LDOS) of graphene and boron nitride on Cu(111) averaged over the plane parallel to the 2D material as a function of STM tip height ${\varvec{h}}$ (Left two columns). The LDOS is decomposed into Cu surface (red), Cu bulk (yellow), and 2D material (graphene or boron nitride) (black). The Cu bulk and graphene states are hybridized at $\left| {{\varvec{E}} - {\varvec{E}}_{{\varvec{F}}} } \right| >$ 1 eV as shown in the yellow-black color gradients. Constant-height (${\varvec{h}}$) current maps at a bias voltage of 0.5 V (Right two columns). Each image is scaled by its average value of integrated LDOS (${\varvec{\rho}}_{{{\text{av}}}}$). The black, blue, and magenta open circles represent the positions of the carbon, boron, and nitrogen atoms, respectively.

LDOS of graphene on Cu(111) at $h =$ 2 Å shows a step-like rise at − 0.55 eV and a V-shaped dip at − 0.29 eV corresponding to the onset of the Cu surface state and the Dirac point of the electron-doped^33^ graphene, respectively. The former is still observable at larger $h$, while the latter is unresolvable for $h \ge$ 3 Å, since the contribution of graphene decays much faster than that of Cu. For a large gap insulator BN on Cu(111), there are no BN states near the Fermi level, so the LDOS is entirely from the Cu(111) surface, which is hardly different from the bare Cu(111) surface.

At a moderate distance between tip and 2D material ($h \ge$ 3 Å), the tunneling current is dominated by substrate states. The most striking finding in this distance range is that $\rho_{{{\text{av}}}}$, which is proportional to the tunneling current, is larger for BN on Cu(111) than for graphene. This means that the insulating BN appears to be more conductive than graphene under the same experimental conditions. Our finding enables the understanding of seemingly puzzling phenomena in the STM community. For example, a lateral heterostructure of graphene and 2D BN on a Cu substrate^30^ shows a discrepancy between the STM topography and actual atomic geometry^31^. Our results shed light on why the measured topography of BN looks much higher (> 0.3 Å) in spite that the calculated 2D material-Cu distances ($d$) are similar for graphene and BN. Moreover, we confirm that the lattice structures (hexagonal contrasts) reflected in the topography of both graphene and BN do not guarantee the contributions of the 2D material, but are only the substrate states scattered by the 2D material.

The above results can be rationalized by examining the individual wavefunctions that constitute the tunneling current. We will first show the fast decay rate of graphene states is attributed to large Bloch wavevectors in the given energy range (bias voltage) from the following analysis. The wavefunction in the vacuum is entirely determined by its energy and planar boundary conditions. Explicitly, the Bloch wavefunction $\psi \left( {x,y,z} \right)$ can be written as in 2D Fourier-transformed form as $\psi \left( {x,y,z} \right) = e^{{i\left( {k_{x} x + k_{y} y} \right)}} u_{{\varvec{k}}} \left( {x,y,z} \right)$ where $u_{{\varvec{k}}} \left( {x,y,z} \right) = \mathop \sum \limits_{{{\varvec{G}}_{\parallel } }} a_{{{\varvec{G}}_{\parallel } }} \left( z \right)e^{{i\left( {G_{x} x + G_{y} y} \right)}}$, ${\varvec{k}} = \left( {k_{x} ,k_{y} } \right)$ is a Bloch wavevector parallel to the surface in the *xy* plane, ${\varvec{G}}_{\parallel } = \left( {G_{x} ,G_{y} } \right)$ the 2D reciprocal lattice vector and $a_{{{\varvec{G}}_{\parallel } }}$ is the coefficient of Fourier component ${\varvec{G}}_{\parallel }$. We then solve the Schrödinger equation in the vacuum $\left( { - \frac{{\hbar^{2} }}{2m}\nabla^{2} + V_{vac} } \right)\psi = E\psi$, satisfying two boundary conditions: (1) in the plane $z = z_{0}$, $\psi \left( {x,y,z_{0} } \right) = e^{{i\left( {k_{x} x + k_{y} y} \right)}} \mathop \sum \limits_{{{\varvec{G}}_{\parallel } }} a_{{{\varvec{G}}_{\parallel } }} \left( {z_{0} } \right)e^{{i\left( {G_{x} x + G_{y} y} \right)}}$ and (2) at the distance far from the surface, the wavefunction should completely diminish, $\psi \left( {x,y,z \to \infty } \right) = 0$. This gives the solution $a_{{{\varvec{G}}_{\parallel } }} \left( z \right) = a_{{{\varvec{G}}_{\parallel } }} \left( {z_{0} } \right)e^{{ - \kappa \left( {z - z_{0} } \right)}}$ with $\kappa^{2} = {\varvec{k}}^{2} + {\varvec{G}}_{\parallel }^{2} + \frac{2m}{{\hbar^{2} }}\left( {V_{vac} - E} \right)$. By defining the effective planar kinetic energy $E_{{2{\varvec{D}}}} = \frac{{\hbar^{2} }}{2m}({\varvec{G}}_{\parallel }^{2} + {\varvec{k}}^{2} )$, the decay rate can be written as follows:

1 $$ \kappa = \sqrt {\frac{2m}{{\hbar^{2} }}\left( {V_{vac} - \left( {E - E_{2D} } \right)} \right)} $$

This is the decay constant of the incident 1D wave with energy $E - E_{{2{\varvec{D}}}}$ at the potential height of $V_{vac}$. Asymptotically, the decay constants of 2D states are determined by the smallest Fourier component ${\varvec{k}} + {\varvec{G}}_{\parallel }$ of the wavefunctions, which decays the slowest. Near the Fermi level (within ~ 1 eV window), the main orbital characters of $u_{{\varvec{k}}} \left( {x,y,z \ge z_{0} } \right)$ are *s* and *pz* for Cu and graphene states, respectively, that $u_{{\varvec{k}}}$ does not have a node in the *xy* plane; the smallest Fourier component becomes a Bloch wavevector ${\varvec{k}}$. This implies that the detailed atomic registry due to rotation, translation, or lattice mismatch between 2D material and substrate, manifested in finite Fourier components, plays a minor role in the magnitude of tunneling current. Nevertheless, at a moderate tip-to-2D material distance, the modulation of the decaying wavefunctions due to the interaction between 2D material and substrate might be observed as a weak Moiré contrast.

Figure 3a shows the plane-averaged local potential as a function of the distance from the surface (dashed line) and the LDOS of four representative states (solid lines) indicated in Fig. 1d, namely the bottom surface (green), top surface (black), and bulk (blue) state of Cu(111) at ${\Gamma }$, and the graphene state at the Dirac point (red). The LDOS profiles shown in the black dotted rectangle of Fig. 3a are magnified in Fig. 3b with a logarithmic axis (thick lines), from which decay rates can be extracted. These values compare very well with the decay rate from the analytical expression in Eq. (1). For the bare Cu(111) surface, the calculated workfunction is $\phi \equiv V_{vac} - E_{F} =$ 4.84 eV and the energy of the surface state at the G point is located at $E - E_{F} =$ − 0.68 eV. When graphene (BN) is on Cu(111), a surface dipole layer forms, lowering the vacuum level by 1.04 (1.14) eV. Note that the dipole layer is originated from the pushback of the electron tail into Cu^34,35^ and the charge transfer from Cu(111) to graphene tends to decrease the shift. Graphene on Cu presses the top surface states of the substrate, leading to an increase in the energy of the top surface states by 0.13 eV at ${\Gamma }$. In summary, $V_{vac} - E$ of graphene on Cu(111) system becomes 5.52, 4.35, 5.19 and 4.09 eV for bottom surface, top surface, bulk state of Cu(111) and Dirac point of graphene, respectively. As mentioned above, the smallest Fourier components of the four states are the same with their Bloch wavevectors, so the effective planar kinetic energy ($E_{{2{\varvec{D}}}}$) vanishes for Cu states and is 10.74 eV $\left( { = \frac{{\hbar^{2} }}{2m}{\varvec{K}}^{2} } \right)$ for graphene states. The decay parameters of LDOS’s calculated from Eq. (1) become 2 $\kappa =$ 2.407, 2.139, 2.334 and 3.946 Å^-1^ and are shown in Fig. 3b with dashed lines that agrees very well with DFT calculations.

**Figure 3:**
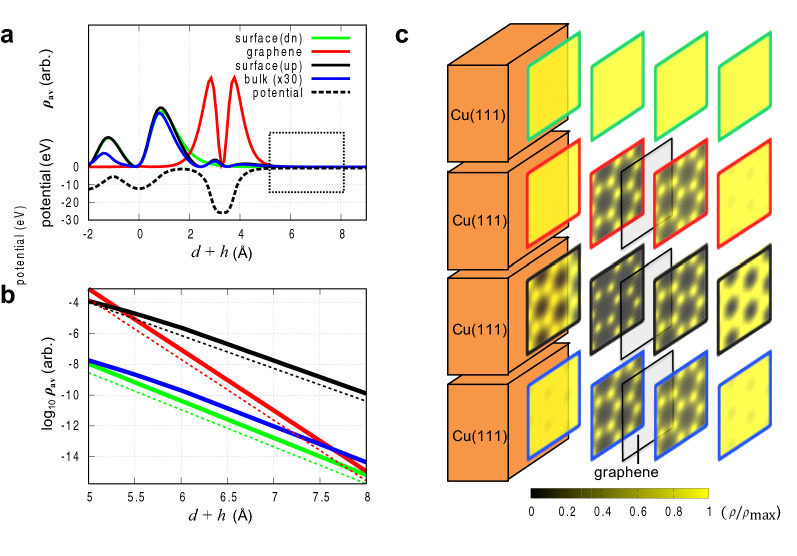
Vacuum-decaying wavefunction tail analysis. (**a**) Plane-averaged LDOS (${\varvec{\rho}}_{{{\text{av}}}} )$ (solid lines) of the four representative states identified in Fig. 1d and the local potential (${\varvec{V}}$) of Gr on Cu(111) (dashed line) as a function of distance from the top surface of the substrate (${\varvec{d}} + {\varvec{h}}$). (**b**) Magnified view of the LDOS’s of the black dotted rectangle in (**a**), comparing the DFT result (solid lines) with the result of the analytical formula (Eq. 1) (dashed lines). (**c**) Cross-sectional view of LDOS’s in real space for ${\varvec{d}} + {\varvec{h}} =$ 1.94, 2.96, 3.98, and 5.00 Å from the lefts to the rights. LDOS’s are normalized with their maximum values in the plane.

We have shown that the Bloch wavevectors of the surface wavefunctions determine the decay rates into the vacuum and that the large Bloch wavevectors of the graphene states cause their electronic structure buried by Cu states. We can assume that this also occurs on other noble metal surfaces or on other crystallographic orientations without surface states such as Cu(100), given that the decay rate is related only to the energy and Bloch wavevectors of the states. In general, the intrinsic electronic structures of 2D materials on a metal substrate can only be observed if they exhibit small planar Bloch wavevectors at the given bias voltage. For graphene, the states between − 7.5 and − 2 eV from the Fermi level are the case, and they are well reflected in the LDOS as demonstrated in Sect. 2 of Supplementary information. Some semiconducting transition metal dichalcogenides such as monolayer H-MoTe_2_ may have similar properties with graphene (most states around conduction band minimum or valence band maximum have large in-plane crystal momentum), but due to the thickness of them (3 atomic layers), the dominant contribution of substrate states seems to be not so drastic.

While the substrates states dominate in the tunneling current, the STM topography resembles the structure of the 2D material on the substrate. This is due to the underlying potential of the 2D material that leads to the in-plane variations of penetrating states from the substrate. Graphene, as an example, generates a spherical potential that is hexagonally arranged at the atomic sites. This potential modulates the penetrating substrate states of Cu, and amplifies them near the potential minimum generated by the 2D material (graphene). This enhancement of the substrate states (black and blue solid lines) is clearly seen in Fig. 3a. In Fig. 3c, the LDOS variations of the four representative states in Fig. 3a are observed in the plane, where the 2D cross-sectional view of the LDOS’s are scaled by their planar maxima $\psi \left( {x,y,z = z_{p} } \right)^{2} /{\text{max}} \psi \left( {x,y,z = z_{p} } \right)^{2}$ at $z_{p} =$ 1.94, 2.96, 3.98 and 5.00 Å. The black-to-yellow color gradation corresponds the change from 0 to 1. The Cu states (green/black/blue) become spatially homogeneous (${\varvec{G}}_{\parallel } = 0$ is dominant) as departing from the surface $(z_{p} =$ 1.94 Å), while in the presence of the 2D material, they are strongly enhanced at each atomic site, as in $z_{p} =$ 2.96 and 3.98 Å cross sections. Away from the 2D material, the nonzero ${\varvec{G}}_{\parallel }$ component decreases rapidly, and ${\varvec{G}}_{\parallel } = 0$ becomes dominant again ($z_{p} =$ 5.00 Å), but there still remains a small nonzero planar variation in density. In contrast to the Cu states, the slowest decaying component is ${\varvec{K}}$ for graphene (red), so the spatial variation itself lasts for a long decaying length, but the rapid decay of the states makes their contribution to the spatial variation of the topography negligible.

Next, we discuss the implication for STM measurements of the substrate states that last longer than the 2D material states as they decay into the vacuum. One of the most noticeable consequences of prolonged substrate states is a topography inversion in STM measurements, where the measured topography of a 2D material on a substrate does not correspond to its actual geometric corrugation and even it is reversed, i.e., a peak looks like a valley and vice versa. As an example, Fig. 4 presents the calculated constant-current topographies of graphene (a) and BN (b) when their distance from the Cu substrate *d* increases slowly, and compares them with the actual changes in the distance profile (red dashed lines). Here, the bias voltage is set to be 0.5 V and the topographies (black solid lines) are drawn for five selected current settings corresponding to $\rho_{{{\text{av}}}} =$ 15, 7.5, 3.8, 1.9, and 0.9 (unit: 10^–5^ e/Å^3^).

**Figure 4:**
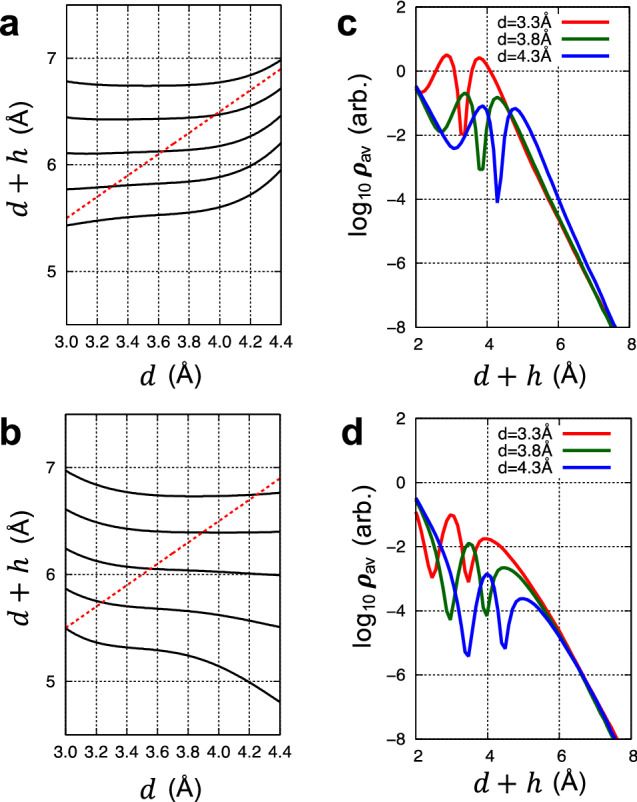
Simulated STM topography of graphene and boron nitride on Cu surface. Constant-current STM topographies of (**a**) graphene and (**b**) BN on Cu(111) when their distance from the Cu substrate ${\varvec{d}}$ increases slowly. The bias voltage is set to be 0.5 V, and topographies (black lines) are drawn for five selected set currents corresponding to ${\varvec{\rho}}_{{{\text{av}}}} =$ 15.0, 7.5, 3.8, 1.9, and 0.9 (unit: 10^–5^ e/Å^3^) from the bottom to the top. They are compared with the actual changes in *d* (red dashed lines). For ${\varvec{d}}$ = 3.3, 3.8 and 4.3 Å, the current values averaged over the plane parallel to the 2D material are plotted in case of (**c**) graphene and (**d**) boron nitride. Here, the current near the surface contains both graphene and Cu states. On the other hand, with increasing *d*, the graphene states disappear completely, so that the current at a large distance consists exclusively of Cu states, which decrease monotonically with ${\varvec{d}}$.

For graphene, the apparent height variation is much less than the actual geometric variation. For $d$ = 3.3, 3.8 and 4.3 Å, we plotted $\rho_{{{\text{av}}}}$ in Fig. 4c. The integrated LDOS around the graphene (two peaks) originates from both the intrinsic graphene states and Cu states. Because the amount of graphene states does not depend on $d$, the decreasing integrated LDOS with increasing $d$ is due to the less enhanced Cu states. As we fix the height of the STM tip, the contribution of graphene to the tunneling current is large at smaller $h$ (larger $d$), while the contribution of Cu becomes smaller. Due to the opposite $d$-dependence of graphene and substrate states, they tend to cancel each other and the apparent STM topography becomes flattened. For BN, the gradients of the STM topography with increasing *d* are mostly negative, which means that the apparent height is even the opposite of the actual atomic geometry. The integrated LDOS around BN comes entirely from Cu states, so the asymmetry of the two peaks, which is the distinguishing feature between Cu states and $\pi$-orbital states of the 2D material, is more pronounced than in graphene. The $d$-dependence of the integrated LDOS shows a monotonic behavior, as shown in Fig. 4d, and a clear topography inversion are manifested.

Finally, we explain the origin of the large tunneling current of an insulator, which is larger than that of a semimetal as graphene at a large distance ($$h \ge 3$$ Å). Although the tunneling current in this distance range is mainly determined by the enhancement factor of the substrate states, many material-specific factors play a role. These include atomic species, atomic density, or surface dipole strength, which necessitate first-principles atomistic modeling for quantitative analysis. Figure 5 compares the integrated LDOS (up to 0.5 V) of graphene and three insulating layers (BN, Ar monolayer registered at hollow sites of Cu(111) and $$\left( {\sqrt 7 \times \sqrt 7 } \right){\text{R}}19.1^\circ$$ structure of benzene monolayer) on Cu(111) with $$d$$ = 3.3 Å. The bottom row shows integrated LDOS at $$ h$$ = 1 Å, and line profiles (dotted red lines) at $$h$$ = 1–4 Å are plotted along the red lines in the image. With the exception of graphene, where 2D material contributions are substantial at small $$h$$, the decay rates for the three insulating layers are similar (~ 0.1 Å^-1^), while the relative magnitudes are material specific. We also confirm that topography inversion occurs for Ar monolayer by comparing the integrated LDOS’s of two Ar monolayers (third column) with $$d$$ = 3 Å (green dotted lines) and $$d$$ = 3.3 Å (red dotted lines).

**Figure 5:**
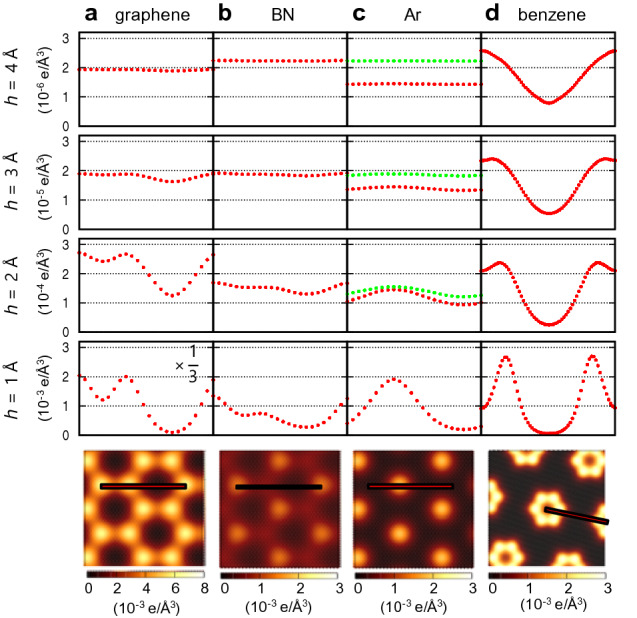
Integrated LDOS of various 2D materials on Cu surface. Integrated LDOS at $h$ = 1 Å (bottom row) and $h$-dependent line profiles (red dotted lines) along the red lines in the image of graphene (**a**), BN (**b**), Ar monolayer (**c**), and benzene monolayer (**d**) located at 3.3 Å above Cu(111) under 0.5 V bias. The Ar atoms are located on the hollow sites of Cu(111) and the benzene molecules form an $\left( {\sqrt 7 \times \sqrt 7 } \right){\text{R}}19.1^\circ$ adsorption structure. The size of image is 6 Å × 6 Å for **a**–**c** and 12 Å × 12 Å for **d**. The green dotted lines in **c** denotes the line profile of the Ar monolayer located at 3.0 Å above Cu(111).

In conclusion, using density functional theory calculation and analytical modeling, we have showed the dominant role of penetrating metal substrate states for atomically thick materials and its consequences such as a topography inversion. Thin materials are prone to develop well-ordered nanoscale corrugations geometrically (nanomesh) or electronically (Moiré pattern) that the corrugation design has been suggested as a new control knob modifying the electronic and chemical properties of parent materials. For those materials, our findings can not only reconcile apparent contradictions between STM topography and atomic structures obtained with first-principles calculations or atomic force microscopy but also help decoupling of intrinsic 2D material properties from the substrate effects.

## Method

Our calculations and analysis are based on the first-principles density functional theory (DFT) employing the Perdew–Burke–Ernzerhof exchange–correlation functional^36^ and the projector augmented wave method for ionic potentials^37^ as implemented in the Vienna Ab Initio Simulation Package^38^. The energy cutoff of planewave basis is 400 eV. Within the Tersoff-Hamann approximation^32^, STM topographies are obtained by the real space summation of wavefunctions in the specific energy range and the wavefunctions are densely sampled (192 × 192 points for 5.387 Å^2^ of 2D cell or denser) in the 2D Brillouin zone. For a standard energy cutoff setting of planewave basis (< 600 eV), the exponentially decaying tails of ~ 3 Å above the 2D material are overwritten by numerical noise. To overcome this technical issue, analytical extrapolations are applied to the wavefunctions to achieve accurate LDOS. Details can be found in the Sect. 3 of Supplementary Information.

## Supplementary Information

Supplementary Information.

## Data Availability

All data generated or analysed during this study are included in this published article and its supplementary information files.
