# Supplementary material for: Topography inversion in scanning tunneling microscopy of single-atom-thick materials from penetrating substrate states
Source: Sci Rep. 2022 May 5;12:7321. doi: 10.1038/s41598-022-10870-0 (PMC9072348; doi:10.1038/s41598-022-10870-0)
Supplement: Supplementary file 1 — Supplementary Information. [file 41598_2022_10870_MOESM1_ESM.docx]

**Supplementary Information**

Changwon Park^1,*^ and Mina Yoon^2^

*^1^Korea Institute for Advanced Study, Hoegiro 85, Seoul 02455, Republic of Korea*

*^2^ Materials Science and Technology Division, Oak Ridge National Laboratory, Oak Ridge, TN 37831, U.S.A.*

**1. Criteria for wavefunction character**

All wavefunctions in our STM simulations were characterized by their spatial distributions. We defined three volumes; (1) bulk region (B): $h_{lo}-1 Å<z<h_{up}+1 Å$, (2) surface region (S): $h_{up}+1 Å<z<h_{up}+2 Å$ and $h_{lo}-2 Å<z<h_{lo}-1 Å$, (3) sample region (2D): $z<|h_{2D}-2.3Å|$, respectively. Here, $h_{up(lo)}$ and $h_{2D}$ are positions of uppermost (lowermost) Cu plane and graphene (boron nitride). For $h_{2D}=3.3 Å$, Fig. S1 (a)-(d) show squares of plane-averaged wavefunctions (red lines) and their charge integrations (blue lines) for prototypical wavefunction character of (a) graphene, (b) surface state, (c) s- and d-orbital hybridized Cu bulk state and (d) d-orbital Cu bulk state. Three defined volumes are colored in green, red and blue, respectively. Charge integration inside each volume ($q$) is used for wavefunction character identifier for the following criteria; (1) $q_{B}\geq0.92$ : bulk state (2) $q_{B}<0.92$ and $\frac{q_{S}}{q_{S}+q_{2D}}>0.35$ : surface state (3) $q_{B}<0.92$ and $\frac{q_{S}}{q_{S}+q_{2D}}\leq0.35$ : graphene or graphene-bulk hybridized state. In Fig. S1(e), the wavefunctions in energy band are colored in green, red and blue according to the criteria (1)-(3), respectively. For $E>E_{F}+1 eV$ and $E<E_{F}-1.5 eV$, graphene states begin to hybridized with bulk states as shown in $q_{B}$- energy plot of Fig. S1 (f). Band wavefunctions satisfying criteria (2) and (3) are shown in Fig. S1 (g) as red and blue dots. The clear separation confirms $\frac{q_{S}}{q_{S}+q_{2D}}$ is a good identifier.

i

Figure S1. Prototypical wavefunctions of (a) graphene, (b) Cu surface state, (c) *s*-*d* hybridized Cu bulk state and (d) Cu *d* bulk state. $h$ denotes distance from Cu surface and graphene in at $h$ = 3.3 Å. Bulk, surface and graphene regions (see text) are colored in green, red and blue, respectively. Red lines are charge density of wavefunction and blue lines are integrated charge density. (e) band structure of graphene on Cu(111). Wavefunctions are characterized as graphene (blue), Cu bulk (green) and Cu surface (red) states according to a given criteria (see text). Wavefunctions are arranged in (f) energy - $q_{B}$ and (g) energy - $\frac{q_{S}}{q_{S}+q_{2D}}$ space.

**2. Local density of states of graphene on Cu(111) for wider energy range**

Figure S2(a) is the band structure of graphene 3.3 Å above Cu(111). Small *k* states of graphene are in the range between -7.5 eV and -2 eV and LDOS of graphene on Cu(111) at $h$ = 4 Å in Fig. S2(b) is similar with that of freestanding graphene at $h$ = 4 Å in Fig. S2(c) in this range. The graded color indicates hybridizations with s-orbital state of Cu. In fact, LDOS of graphene on Cu(111) at $h$ = 4 Å is similar with that of freestanding graphene added with features of scaled LDOS of Cu(111) at 2 Å above surface Cu atom in Fig. S2(d) if we include minor effect from small energy shift and rearrangement by hybridizations. Because Cu states with large k already decay away at 2 Å above the Cu surface, Fig. S2(d) is mostly composed of small *k* states those are largely enhanced by graphene. Though the enhancements depend on the spatial distribution of wavefunction in general, the dependency seems to be small for this case. As a result, considering the weakly interacting nature, LDOS of graphene on Cu(111) are understood as these enhanced Cu states are simply added to LDOS of freestanding graphene.

Figure S2. (a) band structure of graphene on Cu(111) (black) for $d$ = 3.3 Å. The band structures of Cu(111) (green) and graphene (red) are calculated separately and plotted over with appropriated energy shift to match both bands. (b) plane-averaged LDOS of graphene on Cu(111) at $h$ = 4 Å. Red, yellow and black corresponds wavefunction character with Cu surface, Cu bulk and graphene. The mixed states between graphene and Cu bulk are represented as a graded color. (c) LDOS of freestanding graphene at $h$ = 4 Å. (c) 1/200 scaled LDOS of Cu(111) at 2 Å above topmost Cu atoms.

**3. Extrapolation method of decaying wavefunction**

According to Bardeen’s formulation [1], tunneling current $I_{t}$ between STM tip and sample for a bias voltage $V$ can be written as

$$I_{t}=\frac{4\pi e}{\hbar}\int_{0}^{eV} d{\varepsilon\rho}_{T}(E_{F}+\varepsilon) \rho_{S}\left( E_{F}-eV+\varepsilon\right) |M\left( \varepsilon\right)|^{2}$$

where $\rho_{T(S)}$ is density of states of tip (sample) and $M\left( \varepsilon\right)$ is tunneling matrix element. $|M\left( \varepsilon\right)|^{2}$ can be calculated as a double summation over all tip and sample wavefunction with energy$\varepsilon$ as

$|M\left( \varepsilon\right)|^{2}=\frac{\hbar^{2}}{2m}\sum_{\mu\nu} |\int dxdy\left( \psi_{\mu}^{S}(\varepsilon)\frac{\partial}{\partial z}{\psi_{\nu}^{T}(\varepsilon)}^{*}-{\psi_{\nu}^{T}(\varepsilon)}^{*}\frac{\partial}{\partial z}\psi_{\mu}^{S}(\varepsilon) \right)|^{2}$.

The integrations are done at the plane of tip-sample barrier region where the potential is assumed to be uniform.

As the equation suggests, tunneling current is determined from the exponentially decaying tails of wavefunctions. The barrier region cannot be absolutely measured in experiments, but is expected to be reside 4-7 Å above the sample surface. Unfortunately, for standard settings of first-principles calculations (kinetic energy cutoff < 600 eV for planewave basis), exponentially decaying tails over 3 Å above the sample is too small to be distinguished from zero and numerical noise start to overwrite them as shown in the lower panels of Fig. S3 where unphysical negative charge density and oscillations occur.

To overcome this difficulty, the calculated noisy wavefunctions at vacuum regions $\psi(x,y,z>z_{0})$ were replaced by extrapolated ones from $\psi(x,y,z_{0})$ with a plane $z=z_{0}$. Specifically, $z_{0}$ is chosen to be 2.7 Å above the graphene (boron nitride) to satisfy two conditions; (1) artificial oscillation amplitude of wavefunctions tail due to finite planewave basis is negligible and (2) the potential variation at $z>z_{0}$ (vacuum region) is less than 0.1 eV. Once $z_{0}$ is determined, vacuum level $V$ is set to the average potential of extrapolated region and calculated wavefunction at $z=z_{0}$ with eigenvalue $E$ serves as two-dimensional boundary condition $\psi\left( x,y,z_{0} \right)$. The extrapolated wavefunction $\psi(x,y,z)$ should satisfy the schrödinger equation $\left( -\frac{\hbar^{2}}{2m}\nabla^{2}+V \right)\psi=E\psi$ and has the form $\psi\left( x,y,z \right)=\sum_{\boldsymbol{G}_{\boldsymbol{\parallel}}} a_{\boldsymbol{G}_{\boldsymbol{\parallel}}}(z)e^{i(G_{x}x+G_{y}y)}$ where $\boldsymbol{G}_{\boldsymbol{\parallel}}$ is the reciprocal lattice vector parallel to $z=z_{0}$. The Fourier component $a_{\boldsymbol{G}_{\boldsymbol{\parallel}}}\left( z \right)=a_{\boldsymbol{G}_{\boldsymbol{\parallel}}}(z_{0})e^{-\kappa(z-z_{0})}$ where $a_{\boldsymbol{G}_{\boldsymbol{\parallel}}}(z_{0})$ is two-dimensional Fourier component of boundary condition and $\kappa^{2}={\boldsymbol{G}_{\boldsymbol{\parallel}}}^{\boldsymbol{2}}\boldsymbol{+}\frac{\hbar^{2}}{2m}(V-E)$.

Figure S3. Slices of total charge density (integration over all occupied states) of graphene on Cu(111) surface at 2.7, 3.7 and 4.7 Å above the graphene plane with (upper panels) and without (lower panels) analytic extrapolation using planewave cutoff of 400 eV.

**References**

1. Bardeen, J. Tunnelling from a many-particle point of view. *Phys. Rev. Lett.* **6**, 57 (1961).
